# Supplementary material for: In silico candidate variant and gene identification using inbred mouse strains
Source: PeerJ. 2021 Mar 11;9:e11017. doi: 10.7717/peerj.11017 (PMC7956000; doi:10.7717/peerj.11017)
Supplement: Supplemental Information 1 — Supplementary Table 1: For every strain the number of homozygous variants used in the database as well as the number of low confidence, missing and multi-allelic variants, all of which are excluded from fine-mapping. Supplementary Table 2: Variant consequences and their impact as well as the percentage of variant annotations for every consequence. Overall, the 74,480,058 variants have been annotated with 120,927,856 consequences, so on average every variant has more than one consequence. This table is taken from https://m.ensembl.org/info/genome/variation/prediction/predicted data.html. [file peerj-09-11017-s001.pdf]

Supplement: *In silico* candidate variant and gene identification using inbred mouse strains

Munz *et al.*

Strain	Low confidence	Missing	Multi-allelic	Homozygous
129P2/OlaHsd	2759201	464016	0	71650637
129S1/SvImJ	2104725	485850	987	72282292
129S5SvEvBrd	6291207	782468	726	67799453
AKR/J	2127540	501461	5127	72239726
A/J	2000508	475908	5730	72391708
BALB/cJ	1902476	480064	6463	72484851
BTBR	1803722	424366	4298	72641468
BUB/BnJ	3445878	796218	7377	70624381
C3H/HeH	3107657	797373	6311	70962513
C3H/HeJ	1872635	457199	7565	72536455
C57BL/10J	1041721	72063	564	73759506
C57BL/6NJ	850866	39246	61	73983681
C57BR/cdJ	1616447	308528	4383	72944496
C57L/J	1819112	343481	4151	72707110
C58/J	1589594	288777	3776	72991707
CAST/EiJ	4195487	1189690	69504	69419173
CBA/J	1956792	547516	9055	72360491
DBA/1J	2111015	555761	9648	72197430
DBA/2J	2040804	592720	9552	72230778
FVB/NJ	2236272	401711	9975	72225896
I/LnJ	2247049	553205	13401	72060199
KK/HiJ	2200820	492819	15789	72164426
LEWES/EiJ	5459663	789646	26480	68598065
LP/J	2084208	521306	4361	72263979
MOLF/EiJ	5013644	1241831	102928	68515451
NOD/ShiLtJ	2704953	620641	12373	71535887
NZB/B1NJ	2025638	500590	15537	72332089
NZO/HlLtJ	1941079	451271	15282	72466222
NZW/LacJ	2042271	527620	15595	72288368
PWK/PhJ	4038588	1292408	131599	69411259
RF/J	3244259	751145	9947	70868503
SEA/GnJ	3158470	808689	9411	70897284
SPRET/EiJ	6367082	1801098	483567	66222107
ST/bJ	2075242	443681	12580	72342351
WSB/EiJ	2442184	673958	41658	71716054
ZALENDE/EiJ	2671902	997470	51720	71152762

Supplementary Table 1: For every strain the number of homozygous variants used in the database as well as the number of low confidence, missing and multi-allelic variants, all of which are excluded from fine mapping.

Consequence	SO description	SO term	Display term	Impact	N	Percentage
3_prime_UTR_variant	A UTR variant of the 3' UTR	SO:0001624	3 prime UTR variant	MODIFIER	1060963	0.88
5_prime_UTR_variant	A UTR variant of the 5' UTR	SO:0001623	5 prime UTR variant	MODIFIER	476953	0.39
coding_sequence_variant	A sequence variant that changes the coding sequence	SO:0001580	Coding sequence variant	MODIFIER	276	0.00
downstream_gene_variant	A sequence variant located 3' of a gene	SO:0001632	Downstream gene variant	MODIFIER	12275875	10.15
incomplete_terminal_codon_variant	A sequence variant where at least one base of the final codon of an incompletely annotated transcript is changed	SO:0001626	Incomplete terminal codon variant	LOW	147	0.00
intergenic_variant	A sequence variant located in the intergenic region, between genes	SO:0001628	Intergenic variant	MODIFIER	28350706	23.44
intron_variant	A transcript variant occurring within an intron	SO:0001627	Intron variant	MODIFIER	35716638	29.54
mature_miRNA_variant	A transcript variant located with the sequence of the mature miRNA	SO:0001620	Mature miRNA variant	MODIFIER	800	0.00
missense_variant	A sequence variant, that changes one or more bases, resulting in a different amino acid sequence but where the length is preserved	SO:0001583	Missense variant	MODERATE	247063	0.20
NMD_transcript_variant	A variant in a transcript that is the target of NMD	SO:0001621	NMD transcript variant	MODIFIER	5463950	4.52
non_coding_transcript_exon_variant	A sequence variant that changes non-coding exon sequence in a non-coding transcript	SO:0001792	Non coding transcript exon variant	MODIFIER	2662672	2.20
non_coding_transcript_variant	A transcript variant of a non coding RNA gene	SO:0001619	Non coding transcript variant	MODIFIER	22391917	18.52
splice_acceptor_variant	A splice variant that changes the 2 base region at the 3' end of an intron	SO:0001574	Splice acceptor variant	HIGH	3883	0.00
splice_donor_variant	A splice variant that changes the 2 base region at the 5' end of an intron	SO:0001575	Splice donor variant	HIGH	5834	0.00
splice_region_variant	A sequence variant in which a change has occurred within the region of the splice site, either within 1-3 bases of the exon or 3-8 bases of the intron	SO:0001630	Splice region variant	LOW	94706	0.08
start_lost	A codon variant that changes at least one base of the canonical start codon	SO:0002012	Start lost	HIGH	866	0.00
stop_gained	A sequence variant whereby at least one base of a codon is changed, resulting in a premature stop codon, leading to a shortened transcript	SO:0001587	Stop gained	HIGH	3840	0.00
stop_lost	A sequence variant where at least one base of the terminator codon (stop) is changed, resulting in an elongated transcript	SO:0001578	Stop lost	HIGH	841	0.00
stop_retained_variant	A sequence variant where at least one base in the terminator codon is changed, but the terminator remains	SO:0001567	Stop retained variant	LOW	499	0.00
synonymous_variant	A sequence variant where there is no resulting change to the encoded amino acid	SO:0001819	Synonymous variant	LOW	352392	0.29
upstream_gene_variant	A sequence variant located 5' of a gene	SO:0001631	Upstream gene variant	MODIFIER	11817035	9.77

Supplementary Table 2: Variant consequences and their impact as well as the percentage of variant annotations for every consequence. Overall, the 74,480,058 variants have been annotated with 120,927,856 consequences, so on average every variant has more than one consequence. This table is taken from `https://m.ensembl.org/info/genome/variation/prediction/predicted_data.html`.
